# Vasomotor Reaction to Cyclooxygenase-1-Mediated Prostacyclin Synthesis in Carotid Arteries from Two-Kidney-One-Clip Hypertensive Mice

**DOI:** 10.1371/journal.pone.0136738

**Published:** 2015-08-26

**Authors:** Bin Liu, Zhenhua Li, Yingzhan Zhang, Wenhong Luo, Jiling Zhang, Hui Li, Yingbi Zhou

**Affiliations:** 1 Cardiovascular Research Center, Shantou University Medical College, Shantou, Guangdong, China; 2 Department of Pathology, the Second Affiliated Hospital, Shantou University Medical College, Shantou, Guangdong, China; 3 The Central Laboratory, Shantou University Medical College, Shantou, Guangdong, China; The University of Manchester, UNITED KINGDOM

## Abstract

This study tested the hypothesis that in hypertensive arteries cyclooxygenase-1 (COX-1) remains as a major form, mediating prostacyclin (prostaglandin I_2_; PGI_2_) synthesis that may evoke a vasoconstrictor response in the presence of functional vasodilator PGI_2_ (IP) receptors. Two-kidney-one-clip (2K1C) hypertension was induced in wild-type (WT) mice and/or those with COX-1 deficiency (COX-1^-/-^). Carotid arteries were isolated for analyses 4 weeks after. Results showed that as in normotensive mice, the muscarinic receptor agonist ACh evoked a production of the PGI_2_ metabolite 6-keto-PGF_1α_ and an endothelium-dependent vasoconstrictor response; both of them were abolished by COX-1 inhibition. At the same time, PGI_2_, which evokes contraction of hypertensive vessels, caused relaxation after thromboxane-prostanoid (TP) receptor antagonism that abolished the contraction evoked by ACh. Antagonizing IP receptors enhanced the contraction to the COX substrate arachidonic acid (AA). Also, COX-1^-/-^ mice was noted to develop hypertension; however, their increase of blood pressure and/or heart mass was not to a level achieved with WT mice. In addition, we found that either the contraction in response to ACh or that evoked by AA was abolished in COX-1^-/-^ hypertensive mice. These results demonstrate that as in normotensive conditions, COX-1 is a major contributor of PGI_2_ synthesis in 2K1C hypertensive carotid arteries, which leads to a vasoconstrictor response resulting from opposing dilator and vasoconstrictor activities of IP and TP receptors, respectively. Also, our data suggest that COX-1^-/-^ attenuates the development of 2K1C hypertension in mice, reflecting a net adverse role yielded from all COX-1-mediated activities under the pathological condition.

## Introduction

Cyclooxygenase (COX), which exists mainly as COX-1 and -2 isoforms, metabolizes arachidonic acid (AA) to produce vasoactive prostanoids. Among them, prostacyclin (prostaglandin I_2_; PGI_2_) is a major product produced by the vascular endothelium, acting on smooth muscle PGI_2_ (IP) receptors to mediate vasodilatation and protect vessels from the development of diseases [1–4]. However, studies also indicate that PGI_2_ may activate smooth muscle thromboxane prostanoid (TP) receptors to mediate vasoconstriction and function as an endothelium-derived constrictor factor (EDCF) [5–12]. In fact, the vasomotor reaction to PGI_2_ may be modulated by both TP and IP receptors, and an EDCF-like action of PGI_2_ can result from a limited function of IP receptors, which leads to uncovering of the vasoconstrictor activity derived from a concomitant TP receptor activation [5, 8, 13]. Also, while COX-2 is generally considered as a major mediator of PGI_2_ synthesis [14, 15], an increasing number of studies indicate that COX-1 is a major AA metabolizing enzyme in the vasculature [16–18], which mediates endothelium-dependent contraction [10, 11, 19–21] or relaxation [22–24] under normal physiological conditions. However, the role and pathways of COX-1 in regulating diseased vessels still remain controversial.

Under pathophysiological conditions, including hypertension, the expression of COX-2 (an originally identified inducible isoform) is considered to increase [25, 26] and its metabolism is linked to endothelial dysfunction or development of endothelium-derived vasoconstrictor activities [12, 27–34]. However, in aortas from spontaneously hypertensive rats inhibition of COX-1 has been found to abolish both PGI_2_ synthesis and contraction evoked by endothelial stimulation with agonists, such as ACh [35]. It has been proposed that IP receptors become dysfunctional in hypertension, leading to PGI_2_ acting as an EDCF under the pathological condition [5, 35, 36]. Interestingly, we recently showed that in mesenteric arteries from diabetic mice with increased systemic blood pressure, IP receptors still mediate relaxation [37]. Also, COX-1 inhibition abolished the vasoconstrictor response to ACh under the pathological condition [37]. We considered that in hypertensive arteries, not only COX-1 remains as a major COX form, but also the vasoconstrictor response to PGI_2_ may reflect an overcoming of IP receptor-mediated dilator activity by the effect of TP receptors. On the other hand, a major role for COX-1 in endothelium-dependent contraction of hypertensive arteries in the prior reports has been based on results with inhibitors [35, 37], which could have effects independent of their intended target [38]. It would thus be of interest to determine how genetic COX-1 deficiency (COX-1^-/-^) would influence vasomotor reaction under the pathological condition. Due to the absence of hypertension in diabetic COX-1^-/-^ mice, we were unable to address the issue previously [37]. In addition, it has not yet been clear whether IP receptors mediate dilator activity in hypertensive arteries that show vasoconstrictor response to PGI_2_.

Therefore, in this study two-kidneys-one-clip (2K1C) hypertension was induced to wild-type (WT) C57BL/6 mice or those with COX-1^-/-^. Carotid arteries, where COX-1 mediates endothelium-dependent contraction, and the vasoconstrictor response to PGI_2_ reflects the dilator activity of IP receptors being masked by the effect of TP receptors under normal physiological conditions[20, 22], were isolated for biochemical and/or functional analyses.

## Materials and Methods

### Chemicals and solution

N^ω^-nitro-L-arginine methyl ester (L-NAME), ACh, phenylephrine (PE) and the COX substrate, AA were purchased from Sigma (St Louis, MO). PGI_2_, a stable PGI_2_ analogue and IP receptor agonist iloprost, the COX-1 selective inhibitor FR122047, which has an IC_50_ value of 0.03 or 65 μM for COX-1 and COX-2, respectively [39], the TP receptor antagonist SQ29548, the IP receptor antagonist CAY10441, and the TxA_2_ synthase (TXAS) inhibitor ozagrel, which has an IC_50_ value of 11 nM [40], were bought from Cayman Chemicals (Ann Arbor, MI). L-NAME, PE, ACh, iloprost, AA, and FR122047 were dissolved in distilled water (purged with N_2_ for dissolving AA), while PGI_2_ was dissolved in carbonate buffer (50 mM, pH 10.0). SQ29548, CAY10441, and ozagrel were dissolved in DMSO at 2,000-fold working concentration (the final concentration of DMSO was 0.05/100, v/v).

The compositions of physiological salt solution (PSS; pH 7.4 with 95%O_2_-5% CO_2_) were as follows (in mM): NaCl 123, KCl 4.7, NaHCO_3_ 15.5, KH_2_PO_4_ 1.2, MgCl_2_ 1.2, CaCl_2_ 1.25, and d-glucose 11.5. The 60 mM K^+^-PSS (K^+^) was prepared by replacing an equal molar of NaCl with KCl.

### Mice, induction of 2K1C hypertension, and tissue preparation

All procedures performed on mice were in conformance with the Guide for the Care and Use of Laboratory Animals published by the US National Institutes of Health (NIH Publication No. 85–23, revised 1996) and approved by The Institutional Animal Research and Use Committee of Shantou University (Permit Number: STUMC2012-039 and STUMC2014-095). All surgery was performed under sodium pentobarbital anesthesia, and all efforts were made to minimize suffering of the mice.

C57BL/6 and COX-1^-/-^ mice were obtained as described previously [10]. The surgery to induce 2K1C hypertension was performed using a procedure modified from that reported previously [41]. Briefly, male C57BL/6 or COX-1^-/-^ mice (10–12 weeks) were anesthetized with pentobarbital (100mg/kg, i.p.) and a horizontal incision was made on the right back below the lowest rib. The right renal artery was isolated, cleared of fat with a micro glass rod, and then clipped with a silver clip with a 1.2-mm-wide notch in the middle of the main stem.

On the 28^th^ day after surgery, 2K1C mice with systolic blood pressure (SBP) > 140 mm Hg or an increase of > 20 mm Hg compared to the preoperative value and age-matched C57BL/6 mice (to serve as positive controls for the COX-1-mediated response and to contrast the deviation of pathologies developed in 2K1C mice) were euthanized by CO_2_ inhalation. With the help of a binocular microscope, carotid arteries were isolated and dissected free of adherent tissues for biochemical and/or functional analyses. Also, the heart was cut, cleared of blood and adherent tissues, and weighed for the examination of the heart to body weight ratio. For functional studies, vessels were cut into 1 mm rings as described previously [10, 20].

### Blood pressure measurements

SBP was measured on the day of surgery preoperatively and weekly thereafter, using a non-invasive computerized tail-cuff system (ALC-NIBP, ALCBIO; Shanghai, China). Before BP measurement, mice were accustomed to the tail-cuff blood pressure measurements for 3 days before the day of surgery or 1 day before measurement thereafter. SBP was obtained with 3 consecutive measurements that have a constant value.

### Analyses of vasomotor reactions

Analyses of vascular function were performed as described elsewhere [11, 20]. Briefly, the vascular ring was mounted between two tungsten wires in an organ bath filled with PSS aerated with 95%O_2_-5% CO_2_ and maintained at 37°C. One wire was stationary, whereas the other was connected to an AE801 force transducer (Kronex, Oakland, USA). In some experiments, the endothelium was removed by rotating the vessel ring around the two tungsten wires with the passive tension kept at 100 mg. Thereafter, vessels were stimulated with 60 mM K^+^ every 15 minutes, and the resting tension was adjusted stepwise to an optimal level (~250 mg), at which point the response to 60 mM K^+^ was maximal and reproducible.

To remove the influence of NO, some vessels were treated with the NO synthase (NOS) inhibitor L-NAME (1 mM), under which the response of arteries appears similar to that of eNOS^-/-^ mice [42]. Inhibitors were added 30 min before the vessel was contracted with an agonist, and was kept in the solution throughout the experiment. For control responses, the inhibitor was replaced with the vehicle alone. The response elicited by an agent under baseline conditions was expressed relative to that of 60 mM K^+^, while that during the contraction evoked by PE (at the concentration indicated) was expressed relative to the value immediately prior to the application of the agent.

### Assay of 6-keto-PGF_1α_

The PGI_2_ metabolite 6-keto-PGF_1α_ was measured with an EIA kit [10]. Briefly, after being cut open and rinsed of blood components, carotid arteries (vessels from both sides were pooled for each single experiment) were incubated with PSS at 37°C for 30 min, followed by exposure to PSS (100 μl) and ACh (10 μM) in 100 μl PSS (37°C) for 15 min each. In some experiments, FR122047 (1 μM) was added to the incubating buffer, and was kept in reaction solution throughout the experiment. Thereafter, vessels were taken out, and the reaction solution was diluted with PSS (1:10). Measurement of 6-keto-PGF_1α_ was performed with 50 μl of final diluted solution, using a protocol described in instructions of the manufacturer. The amount of 6-keto-PGF_1α_ was expressed as ng per mg of wet tissue.

### Real-time PCR

Expressions of IP, TP receptors, and β-actin (internal controls) were detected by real-time PCR. Vessel specimens (pooled from 2 mice for each single set of experiments) were cut open and rinsed of blood components, followed by mincing and homogenizing in an ice-cold RNAiso Plus solution (TaKaRa, Dalian, China), using a glass homogenizer. In some experiments, the opened vessel strips were further denuded of endothelium by rubbing with a moistened cotton swab, which was made around one-tip of a micro-dissecting forceps, under a binocular microscope. Total RNA was prepared according to the manufacturer’s instructions. First-strand cDNA was synthesized using total RNA (250 ng) and oligo(dT)15 primers (TaKaRa). The PCR primers for IP, TP receptors, and β-actin were described previously (19). Real-time PCR was performed using a SYBR PrimScript RT-PCR kit (TaKaRa).

### Data analysis

Data were expressed as means ± SEM from n numbers or pools of vessels from different animals. For statistical evaluation, a Student's t-test (unpaired; two tails) was performed to compare two means. When more than two means were compared, a one-way or two-way ANOVA followed by Bonferroni’s post-hoc test was used. P<0.05 was considered to be statistically significant.

## Results

### Effect of COX-1 inhibition on PGI_2_ synthesis and relaxation evoked by ACh

**Fig 1:**
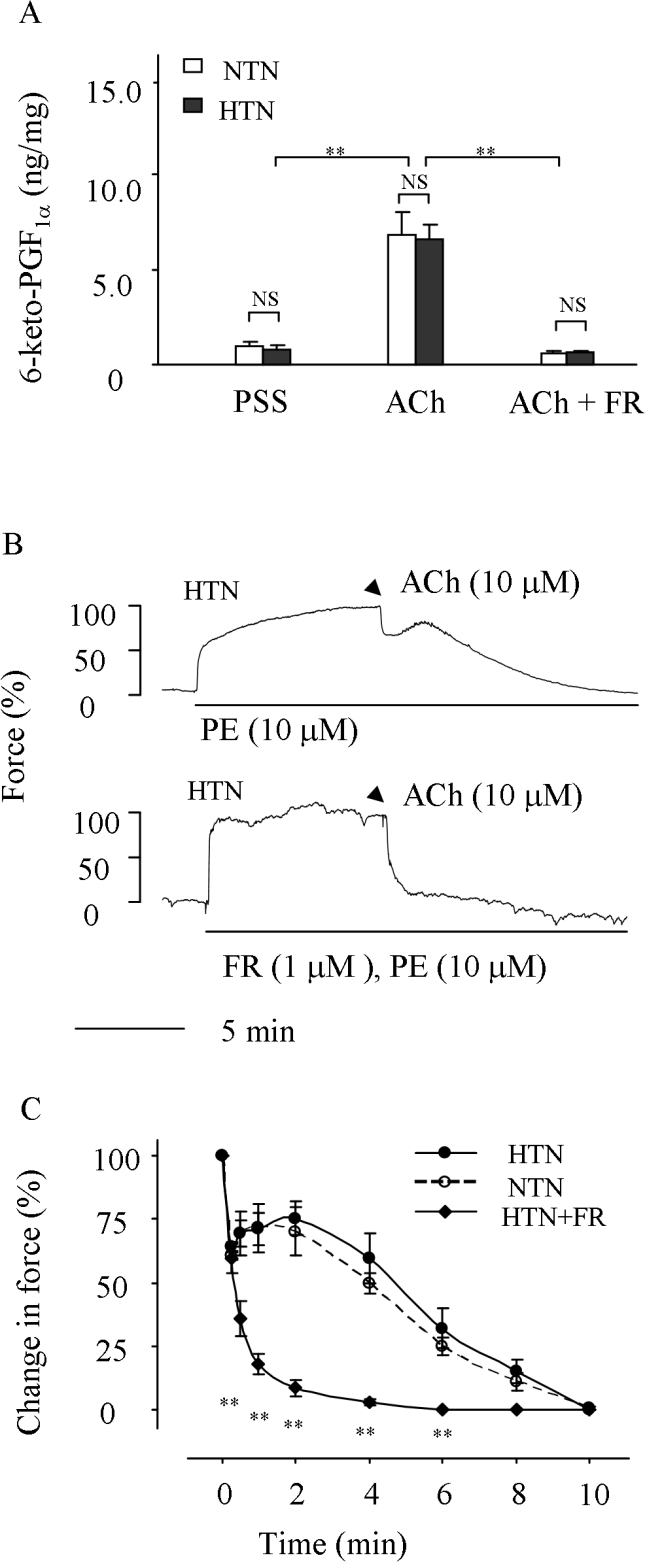
PGI_2_ synthesis and response evoked by ACh in 2K1C hypertensive carotid arteries. ***A*:** detection of the PGI_2_ metabolite 6-keto-PGF_1α_ evoked by ACh with EIA in 2K1C hypertensive (HTN) and control normotensive (NTN) arteries with/out the presence of 1 μM COX-1 inhibitor FR122047 (FR). Values were expressed as mean ± SEM (n = 6 for each). ** P<0.01; NS, not significant. ***B*:** represent traces showing time-courses of the relaxation evoked by ACh (10 μM) in HTN pre-contracted with PE (10 μM) and that obtained in the presence of FR (HTN+FR). ***C*:** summary of the time-course of the relaxation evoked by ACh in HTN as compared to that of NTN or HTN+FR. Values were expressed as mean ± SEM (n = 5 for each); **P<0.01 vs. the value of HTN.

The effect of COX-1 inhibition on the in vitro production of the PGI_2_ metabolite 6-keto-PGF_1α_ and relaxation evoked by the maximal concentration of muscarinic receptor agonist ACh (10 μM) was first examined [35, 42]. As shown in Fig 1A, in hypertensive carotid arteries, ACh caused a >10-fold increase in the production 6-keto-PGF_1α_ compared to resting conditions. However, the addition of selective COX-1 inhibitor FR122047 (1 μM) reduced 6-keto-PGF_1α_ to a level close to resting conditions, similar to results observed in the control normotensive mice. No difference was found in levels of 6-keto-PGF_1α_ between hypertensive and normotensive vessels (Fig 1A).

Also, in hypertensive carotid arteries pre-contracted with 10 μM PE (to yield a sustained contraction of 80–100% that of 60 mM K^+^) the relaxation evoked by ACh (10 μM) was blunted by a biphasic force development (Fig 1B & 1C). Interestingly, FR122047 (1 μM) abolished the biphasic force development and resulted in an enhanced relaxation, which had a tension within 6 min after the application of ACh being significantly lower than that of control hypertensive vessels (Fig 1B and 1C). In addition, we noted that the overall time course of the response evoked by 10 μM ACh in such treated hypertensive vessels was similar to that of nomortensive mice (Fig 1C).

### ACh-evoked response in hypertensive carotid arteries with NOS inhibited

**Fig 2:**
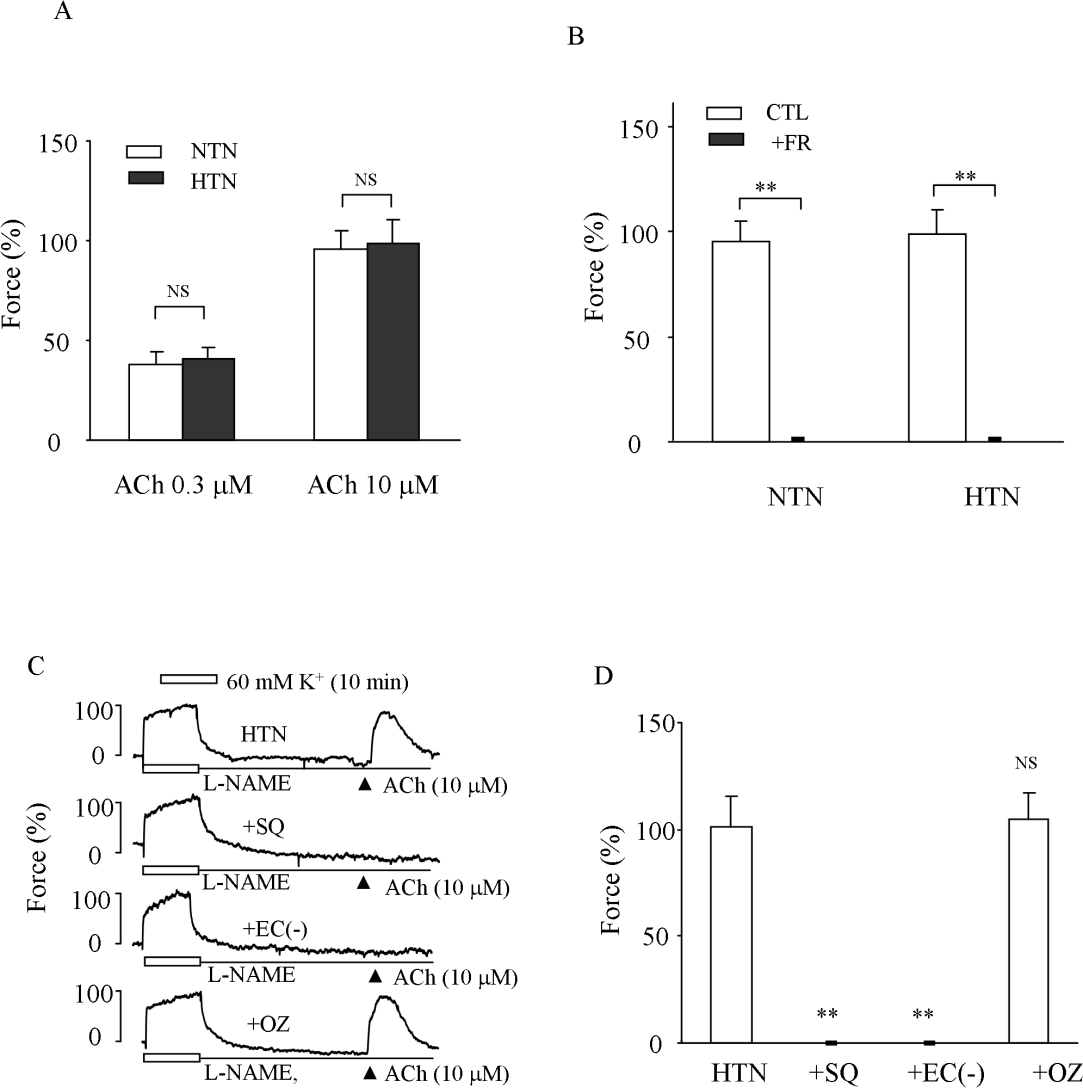
Response to ACh in HTN carotid arteries with NO synthase inhibited. ***A*:** summary of contractions evoked by 0.3 and 10 μM ACh in HTN and NTN arteries treated with 1 mM L-NAME. ***B*:** comparison of contractions evoked by 10 μM ACh in NTN and HTN vessels with those obtained in the presence of 1 μM FR122047 (+FR). ***C*:** representative traces showing the control response evoked by ACh (10 μM) in L-NAME-treated HTN arteries (top), the response obtained in the presence of 3 μM of TP receptor antagonist SQ29548 (+SQ; 2^nd^ trace), and that in vessels denuded of the endothelium (+EC-; 3^rd^ trace) or obtained in the presence of 3 μM TXAS inhibitor ozagrel (+OZ; bottom). ***D*:** summarized results from C. In A, B and D, values were expressed as mean ± SEM (n = 5 for each), **P<0.01; NS, not significant.

The above functional analyses suggest that COX-1 in fact mediates a vasoconstrictor response in hypertensive carotid arteries, which might be of a similar extent to that of normotensive mice revealed previously [10]. To substantiate this, responses evoked by ACh in NOS-inhibited conditions were examined. As shown in Fig 2A, in L-NAME-treated hypertensive arteries either 0.3 or 10 μM ACh evoked contraction under baseline conditions comparable to that of normotensive mice. However, FR122047 (1 μM) abolished the contraction evoked by 10 μM ACh in both normotensive and hypertensive vessels (Fig 2B). Also, the contraction to ACh (10 μM) in hypertensive arteries was removed either by the TP receptor antagonist SQ29548 (3 μM) or by endothelial denudation (Fig 2C & 2D), in a manner similar to that of some other normotensive mouse arteries reported previously [10, 42]. In contrast, the TXAS inhibitor ozagrel (IC_50_: 11 nM) did not show any effect at a concentration of 3 μM (Fig 2C and 2D).

### Response to PGI_2_ in hypertensive carotid arteries

**Fig 3:**
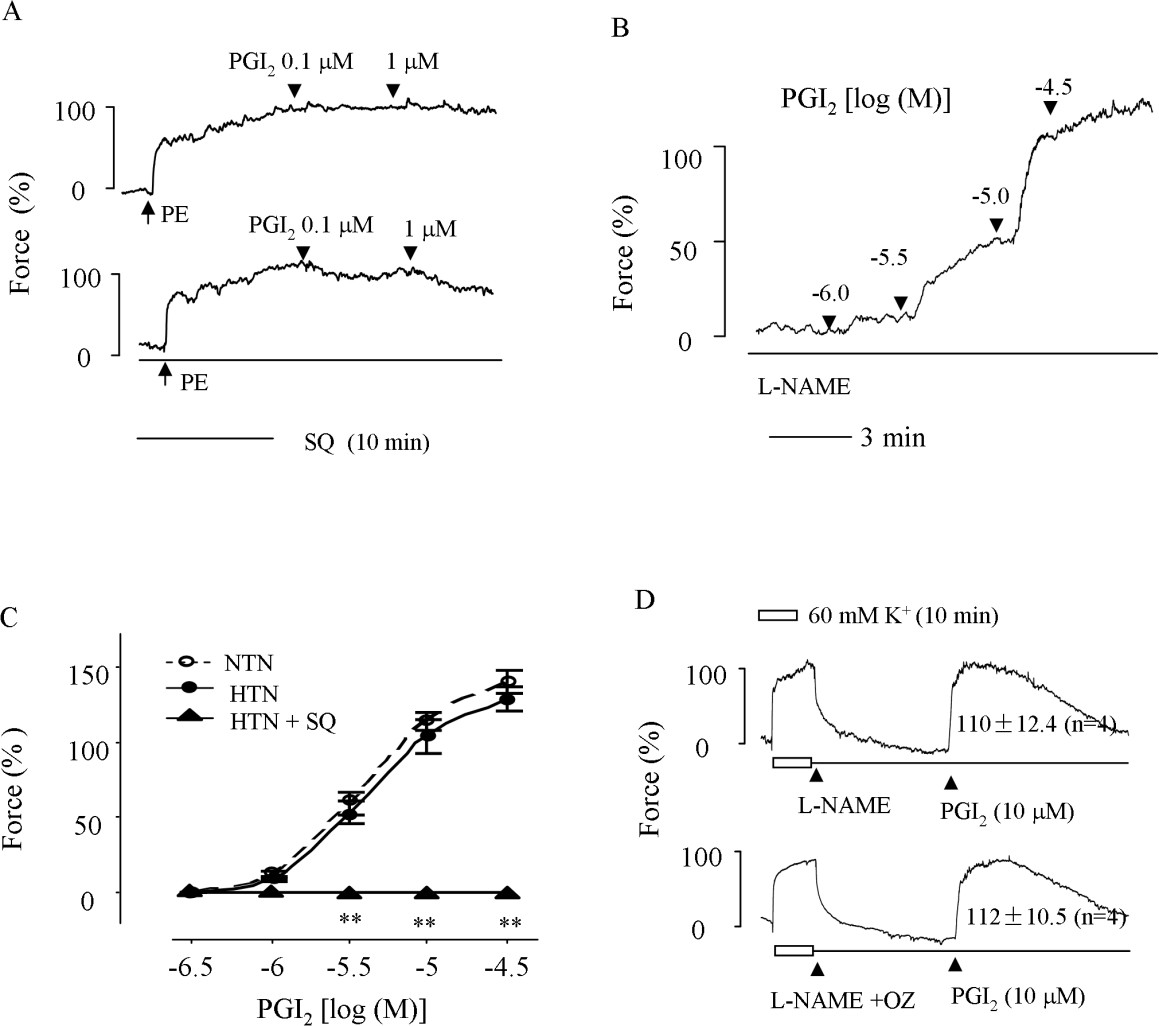
Vasomotor reaction to PGI_2_ in HTN carotid arteries. ***A*:** representative traces showing the response evoked by 0.1–1 μM PGI_2_ in intact (untreated with L-NAME) HTN carotid arteries pre-contracted with 1 μM PE in the absence (top) or presence (bottom) of 3 μM TP receptor antagonist SQ29548 (SQ). ***B*:** a representative trace showing the contraction evoked by PGI_2_ (1–30 μM) in L-NAME-treated HTN carotid arteries. ***C*:** summary of contractions evoked by PGI_2_ (1–30 μM) in L-NAME-treated HTN as compared to those of NTN arteries or those of HTN vessels obtained in the presence of 3 μM TP receptor antagonist SQ29548 (HTN+SQ). Values were expressed as mean ± SEM (n = 5 for each); ** P<0.01. ***D*:** representative traces with summarized values showing contractions evoked by PGI_2_ (10 μM) in L-NAME-treated HTN arteries in the absence (top) or presence (bottom) of 3 μM TXAS inhibitor ozagrel (+OZ). Values were expressed as mean ± SEM (n = 4 for each); P>0.05.

Next, we determined the effect of PGI_2_ on hypertensive carotid arteries. As shown in Fig 3A, even in NOS intact hypertensive carotid arteries pre-contracted with 1 μM PE (to reach 40–60% contraction evoked by 60 mM K^+^, at which point, a dilator activity could be readily detected) PGI_2_ did not evoke any relaxation up to 1 μM. Moreover, in those treated with the NOS inhibitor L-NAME, PGI_2_ (> 1 μM) evoked contraction under baseline conditions, similar to that of normotensive vessels (Fig 3B & 3C). Notably, the TP receptor antagonist SQ29548 not only abolished the contraction evoked by PGI_2_ under baseline conditions (Fig 3C), but also resulted in relaxation (18.2 ± 1.99% and 38.0 ± 5.53% decrease of force vs. 3.2 ± 2.29% and 5.5 ± 3.60% increase of force evoked by 0.1 and 1 μM PGI_2_ in control hypertensive vessels, respectively; n = 5, P<0.01) in response to the agonist in vessels pre-contracted with PE (refer to Fig 3A for representative traces). Again, the TXAS inhibitor ozagrel (3 μM) did not show any effect (P>0.05) on the sub-maximal contraction evoked by 10 μM PGI_2_ (Fig 3D).

### Expressions and functions of TP and IP receptors in hypertensive carotid arteries

**Fig 4:**
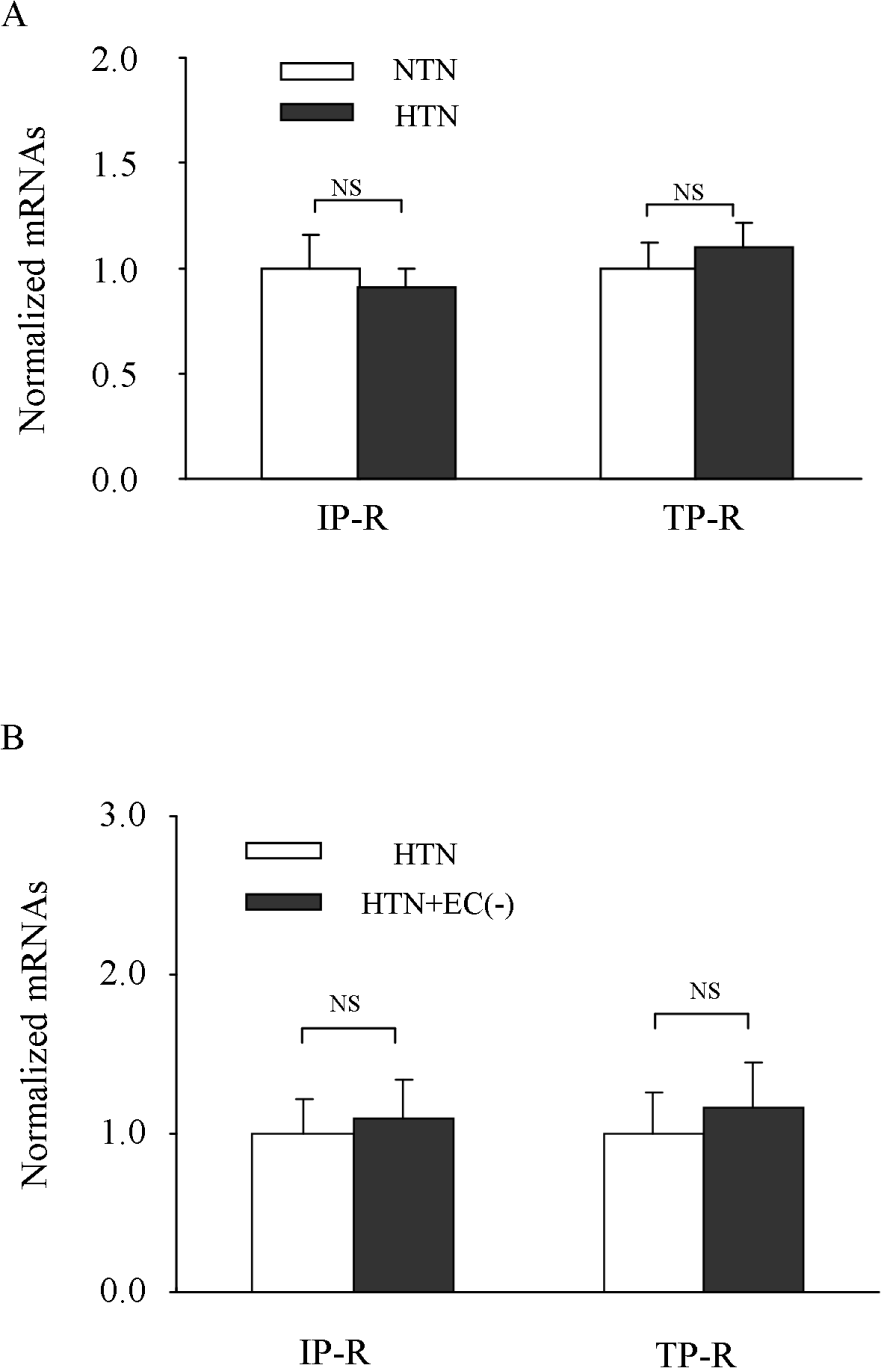
Expressions of IP and TP receptors in HTN carotid arteries. ***A*:** comparison of mRNA levels of IP and TP receptors detected with real-time PCR between HTN and NTN arteries (normalized to β-actin with the average value of NTN vessels assumed as 1.0; n = 6 for each). ***B*:** β-actin normalized levels of IP and TP receptor mRNAs in HTN arteries and those denuded of the endothelium [HTN + EC (-)] with the average value of control HTN vessels assumed as 1.0 (n = 3 for each). Values were expressed as mean ± SEM; NS, not significant.

Since PGI_2_ evoked relaxation after TP receptor antagonism, the expression levels and functions of IP and TP receptors in hypertensive carotid arteries were determined. Real-time PCR showed that amounts of either IP or TP receptor mRNAs were similar between hypertensive and normotensive carotid arteries (Fig 4A). In addition, in hypertensive arteries, mRNAs of both IP and TP receptors were not altered by endothelial denudation (Fig 4B), consistent with a generally proposed major functional presence of these two receptors in medial smooth muscle [36].

**Fig 5:**
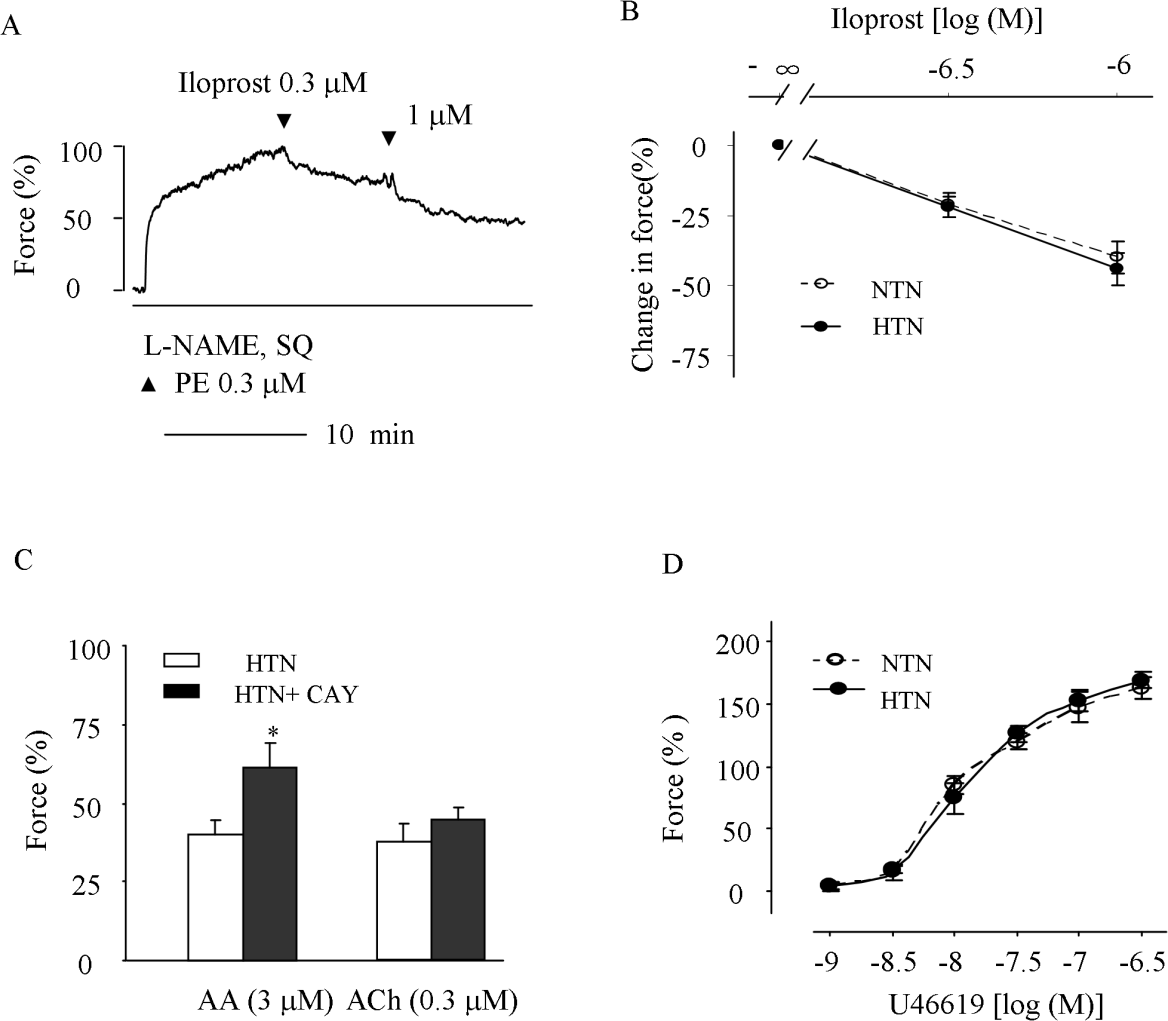
Functions of IP and TP receptors in HTN carotid arteries. ***A*:** a representative trace showing the relaxation evoked by iloprost (0.3–1 μM) in L-NAME-treated HTN arteries pre-contracted with 0.3 μM PE in the presence of 3 μM TP receptor antagonist SQ29548 (SQ). ***B*:** comparison of the relaxation evoked by 0.3–1 μM iloprost (with SQ) in HTN and NTN arteries. ***C*:** effects of IP receptor antagonist CAY10441 (+CAY; 1 μM) on similar extent of contractions evoked by AA (3 μM) and ACh (0.3 μM) in L-NAME-treated HTN arteries. ***D*:** responses evoked by the TP receptor agonist U46619 in L-NAME-treated HTN and NTN arteries. In B, C and D, values were expressed as mean ± SEM. (n = 5 for each); * P<0.05 and ** P<0.01.

At the same time, we noted that after antagonizing TP receptors with SQ29548 (3 μM), 0.1–1 μM iloprost (a stable PGI_2_ analogue and IP receptor agonist, which is less effective than PGI_2_ on TP receptors [11]) evoked relaxation in L-NAME-treated hypertensive carotid arteries pre-contracted with 0.3 μM PE (Fig 5A & 5B), which was similar to that of normotensive mice, and concurring with the NO-independent property of IP receptor-mediated relaxation [22]. In addition, in hypertensive carotid arteries the IP receptor antagonist CAY10441 (1 μM) increased the sub-maximal contraction evoked by the COX substrate AA (3 μM), though it did not show such an effect on a similar response evoked by 0.3 μM ACh (Fig 5C). Also, the contractions evoked by the TP receptor agonist U46619 was similar between hypertensive and normotensive vessels (Fig 5D).

### Effect of COX-1^-/-^ on vasoconstrictor response to AA or ACh

**Fig 6:**
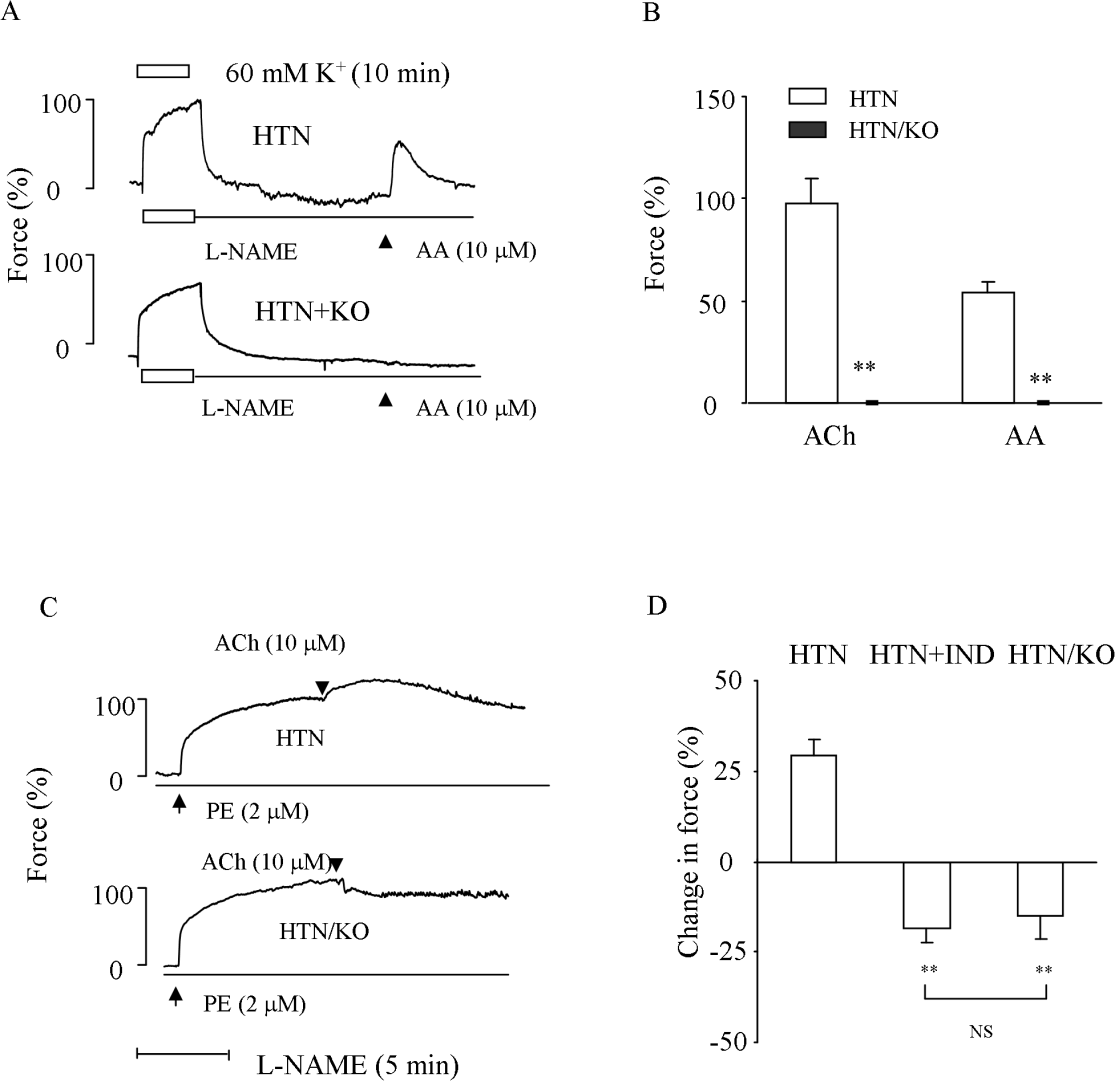
Effect of COX-1^-/-^ on the response to COX substrate AA and/or that evoked by ACh in NOS-inhibited HTN carotid arteries. ***A*:** representative traces showing that contraction evoked by AA in HTN (top) or that in COX-1^-/-^ 2K1C hypertensive vessels (HTN/KO; bottom) arteries. ***B*:** summary of the response evoked by ACh (10 μM) and that to AA (10 μM) in HTN/KO as compared to HTN counterparts. ***C*:** representative traces showing responses evoked by ACh (10 μM) in HTN (top) and HTN/KO arteries (bottom) pre-contracted with PE (2 μM). ***D*:** Summarized results of C together with that obtained with non-selective COX inhibitor indomethacin (10 μM) in WT hypertensive vessels (HTN+IND). In A & C, values were expressed as mean ± SEM (n = 5 for each). **P<0.01 as compared to HTN. NS: not significant.

Lastly, the effect of COX-1^-/-^ on the contraction to the COX substrate AA or that evoked by ACh in 2K1C hypertensive carotid arteries was examined. As shown in Table 1, COX-1^-/-^ 2K1C mice (preoperative SBP: 106 ± 2.4 vs. 109 ± 2.1 mmHg of WT mice, n = 6, P>0.05) developed hypertension; however, their SBP was lower, and the increase of heart mass was not to a significant extent as that of WT counterparts (Table 1). Notably, in L-NAME-treated COX-1^-/-^ 2K1C hypertensive arteries, either the contraction to the COX substrate AA (10 μM) or that to ACh (10 μM) disappeared (Fig 6A & 6B). Moreover, the increase of force evoked by ACh (10 μM) in L-NAME-treated WT hypertensive vessels pre-contracted with PE (2 μM; to reach 80–100% contraction evoked by 60 mM K^+^) was reversed into relaxation in vessels from COX-1^-/-^ 2K1C hypertensive mice, similar to that of WT hypertensive vessels treated with 10 μM of non-selective inhibitor indomethacin (Fig 6C & 6D).

**Table 1:** Hypertension in COX-1^-/-^ 2K1C mice.

	NTN (n = 6)	WT/HTN (n = 6)	KO/HTN (n = 6)
SBP (mmHg)	110.3±1.7	149.7±4.1 **	133.4 ± 3.7 ** ^##^
Heart weight (mg)	108.8±4.4	139.0±10.2 *	116.5±5.4
Heart to body weight ratio (mg/g)	3.78±0.06	5.71±0.33 *	4.76±0.31

NTN: normotensive mice; WT/HTN: wild-type 2K1C hypertensive mice; KO/HTN COX-1^-/-^2K1C hypertensive mice; SBP: systolic blood pressure. Values were expressed as mean ± SEM

* P<0.05

** P<0.01 vs. NTN

##P<0.01 vs. WT/HTN.

## Discussion

In this study, we demonstrated that the muscarinic receptor agonist ACh stimulated a production of the PGI_2_ metabolite 6-keto-PGF_1α_ and an endothelium-dependent contraction in 2K1C hypertensive carotid arteries; both of them were sensitive to COX-1 inhibition. Meanwhile, PGI_2_ was noted to evoke contraction that was reversed into relaxation after TP receptor antagonism. Antagonizing IP receptors enhanced the contraction evoked by the COX substrate AA. Also, we noted that in COX-1^-/-^ 2K1C hypertensive mice, not only was the contraction to ACh or arachidonic acid (AA) abolished, but also the increase of SBP and/or heart mass did not reach an extent seen in WT counterparts. Therefore, COX-1, whose activities altogether appear to adversely influence the development of hypertension, remains as a major form in carotid arteries from 2K1C hypertensive mice, mediating PGI_2_ synthesis that evokes a vasoconstrictor response in the functional presence of dilator IP receptors.

The measurements of 6-keto-PGF_1α_ production clearly indicate that in 2K1C hypertensive carotid arteries, ACh stimulates PGI_2_ synthesis in a manner similar to that of control normotensive mice. Furthermore, FR122047, a COX-1 selective inhibitor, abolished 6-keto-PGF_1α_ production evoked by ACh both in normotensive and in 2K1C hypertensive arteries, suggesting a critical role for COX-1 in PGI_2_ synthesis under normotensive and hypertensive conditions. Also, our functional analyses revealed that FR122047 not only prevented a biphasic force to result in enhanced relaxation to ACh in NOS-intact 2K1C hypertensive carotid arteries, but also abolished a contraction evoked by the agonist in L-NAME-treated vessels under baseline conditions. This further suggests a vasoconstrictor role for COX-1 under the hypertensive condition. It should be noted that the contraction to ACh in hypertensive carotid arteries was similar to that of normotensive mice (which was sensitive to FR122047 as well). Also, concurring with a major expression of COX-1 in endothelium of mouse arteries [10, 43], endothelial denudation removed the contraction to ACh. In addition, we noted that in COX-1^-/-^ hypertensive arteries, not only contractions evoked by ACh and the COX substrate AA disappeared, but also increase of force evoked by ACh in PE pre-contracted conditions was reversed into relaxation as with non-selective COX inhibition in WT hypertensive mice. These results point to that COX-1, whose function remains unaltered, acts as a major form mediating both PGI_2_ synthesis and a vasoconstrictor response in 2K1C hypertensive carotid arteries.

At the same time, we noted that PGI_2_, which caused contraction of the hypertensive arteries, evoked relaxation after TP receptor antagonism. This not only verifies a vasoconstrictor effect of PGI_2_ via TP receptors, but also suggests a dilator role for IP receptors in 2K1C hypertensive carotid arteries where both COX-1 and PGI_2_ mediate contraction. Indeed, in such vessels the relaxation evoked by the stable PGI_2_ analogue iloprost (following TP receptor antagonism) and the mRNA level of IP receptors were comparable to those of normotensive mice. In addition, mRNA levels and contractions evoked by U46619 suggest that TP receptors were also unaltered under the hypertensive conditions. As a result, the vasoconstrictor response to PGI_2_ implies an overcoming of IP receptor-mediated dilator activity by the vasoconstrictor effect of TP receptors, as we previously showed in normotensive conditions [22, 43]. In fact, the amount of PGI_2_ produced by ACh-evoked, COX-1-mediated metabolism is well above its initial level (1 μM) to evoke contraction (1.0 ng/mg 6-keto-PGF_1α_, which equals 2.7 μmol PGI_2_ per kg vessel tissue). In addition, PGH_2_, an intermediate of PGI_2_ synthesis, can activate TP receptors before being converted to PGI_2_ in the medial smooth muscle [35, 43, 44]. Therefore, PGI_2_ synthesis would eventually lead to a vasoconstrictor response and contribute significantly to the contraction evoked by ACh (which was also sensitive to TP receptor antagonism) in 2K1C hypertensive carotid arteries. In contrast, the involvement of TxA_2_, an originally proposed EDCF [36, 42], could be largely excluded by the experiments with TXAS inhibition, which did not reduce the contraction to ACh or evoked by PGI_2_.

Also of interest is that the IP receptor antagonist CAY10441, which enhances contraction but inhibits relaxation to PGI_2_ [5, 22], increased the contraction evoked by the COX substrate AA (which was COX-1-dependent as discussed above). We have previously shown that in mouse arteries, AA stimulates PGI_2_ synthesis [22, 43]. Thus, the above enhancement of AA contraction substantiates the presence of PGI_2_ synthesis in 2K1C hypertensive carotid arteries and implies that under the pathological condition, the native COX-1-mediated AA metabolism activates both TP and IP receptors to result in a net response of contraction. On the other hand, CAY10441 did not increase the contraction to ACh under similar conditions. It has already been known that unlike that of AA, the response evoked by ACh implicates an endothelium-derived hyperpolarizing factor (EDHF)-mediated dilator activity [43], which we also verified by a relaxing response to ACh in NOS-inhibited COX-1^-/-^ 2K1C hypertensive carotid arteries. It should be noted that EDHF mediates relaxation via pathways similar to those of IP receptors [45]. Thus, there exists a possibility that the effect of IP receptor antagonism on the ACh-evoked response is compensated by the redundancy of EDHF-mediated dilation activity. However, this remains speculative; the exact reason(s) for the inability of IP receptor antagonism to enhance the contraction to ACh still requires further investigation.

To date, there has been considerable inconsistency regarding COX isoforms in mediating PGI_2_ synthesis and/or endothelium-derived vasoconstrictor activity under hypertensive conditions [12, 28, 29, 31–33, 35, 46, 47]. An important reason for this could be that some of COX inhibitors used in prior studies might have effects independent of their intended targets [38, 48, 49]. In the present study using C57BL/6 and COX-1^-/-^ mice, our results clearly indicate that COX-1 remains as a major form in 2K1C hypertensive carotid arteries, mediating PGI_2_ synthesis that leads to a vasoconstrictor response as in normotensive conditions. Moreover, our results from SBP and heart mass measurements further suggest that COX-1^-/-^ attenuates the development of 2K1C hypertension in mice. Indeed, a similar beneficial effect of COX-1^-/-^ or COX-1 inhibition has been previously obtained with diabetic mice or a rat model of angiotensin II/salt-induced hypertension, respectively [37, 50]. On the other hand, in 2K1C hypertensive carotid arteries the contraction evoked by ACh was comparable to that of normotensive mice. In addition, COX-1 also mediates TxA_2_ synthesis in platelets, which has been suggested to implicate the pressor response of angiotensin II that plays an essential role in the development of 2K1C hypertension [41, 51]. Also, COX-1 may be linked to oxidative stress, which impairs endothelial function and influences vascular remodeling [36, 52]. Therefore, the attenuation of hypertension in COX-1^-/-^ 2K1C mice could reflect a net adverse role resulting from all COX-1-mediated activities rather than altered endothelium-derived vasoconstrictor activity under the pathological condition.

Also, IP receptors have been previously thought to become dysfunctional, leading to PGI_2_ acting as an EDCF under hypertensive conditions [5, 36]. However, our results showed that IP receptor-mediated dilator function was preserved in the 2K1C hypertensive carotid arteries where PGI_2_ evoked contraction. In some other vascular beds, the dilator function of IP receptors outweighs the effect of TP receptors [22, 23, 53], and hence a vasodilator response to PGI_2_ synthesis would be expected in such vessels even under hypertensive conditions. These results thus further imply a diversity of vasomotor reactions evoked by endothelial COX-1-mediated AA metabolism in hypertension and therefore, TP receptors (which mediate the vasoconstrictor activity of COX-1) rather than COX-1 should be considered as a target for pharmacological intervention of the disorder under clinical conditions.

In summary, our results explicitly demonstrate that COX-1 remains as a major form in 2K1C hypertensive carotid arteries, mediating PGI_2_ synthesis that evokes a vasoconstrictor response resulting from opposing dilator and constrictor activities derived from IP and TP receptors, respectively. In addition, our data suggest that COX-1^-/-^ attenuates the development of 2K1C hypertension in mice, reflecting a net adverse role yielded from all COX-1-mediated activities under the pathological condition.
